# Reproductive Planning and Preconception Counseling in Kidney Disease (REPKID):A Cross-Sectional Survey

**DOI:** 10.1016/j.xkme.2026.101393

**Published:** 2026-05-07

**Authors:** Gwendolyn Lee, Maha Al Baghdadi, Yaquelin Arevalo Iraheta, Lorna Kwan, Christina S. Han, Niloofar Nobakht

**Affiliations:** 1 Department of Medicine, University of California, Los Angeles, CA; 2 Division of Nephrology, Department of Medicine, University of California, Los Angeles, CA; 3 David Geffen School of Medicine, University of California, Los Angeles, CA; 4 Department of Urology, University of California, Los Angeles, CA; 5 Division of Maternal-Fetal Medicine, Department of Obstetrics and Gynecology, University of California, Los Angeles, CA

**Keywords:** Preconception, counseling, kidney disease, pregnancy, multidisciplinary care

## Abstract

**Rationale & Objective:**

Chronic kidney disease (CKD) increases risks of adverse maternal and fetal outcomes. Preconception counseling (PCC) aims to minimize health risks by optimizing prepregnancy health and modifiable risk factors. Although PCC is recommended for all patients, PCC’s utilization and impact among patients with CKD are unknown. This study identified the current utilization and impact of PCC among patients with CKD at an urban academic medical center.

**Study Design:**

This survey-based study evaluated patients’ self-reported demographic characteristics, kidney disease history, PCC exposure and understanding, and interactions with the health care system.

**Setting & Participants:**

Participants included 108 patients assigned female sex at birth and aged 18-45 years at an urban, academic nephrology clinic surveyed between January and May 2024.

**Exposures:**

Exposures of interest included sociodemographic characteristics, clinical CKD presentation, and health care utilization patterns.

**Outcomes:**

The primary outcome was self-reported receipt of PCC. Secondary outcomes included the specialty of the provider offering PCC, specific PCC topics discussed, and patient-reported levels of concern and knowledge.

**Analytical Approach:**

Cross-sectional analysis used χ^2^ or Fisher exact tests to compare respondents who reported they did vs did not receive PCC. Logistic regressions identified characteristics associated with self-reported lack of PCC.

**Results:**

Overall, 74% of respondents reported receiving PCC. PCCs from a maternal-fetal medicine provider were more likely to discuss medication safety, childbearing plans, and maternal and fetal complications. PCC recipients also reported greater concern about fertility but less knowledge about preconception and pregnancy with CKD.

**Limitations:**

Findings may lack generalizability given the single-center, English-speaking population and potential recall bias from self-reported data.

**Conclusions:**

As PCC is recommended for all patients, especially those with a comorbid condition, PCC for patients with CKD remains suboptimal, with 26% reporting no PCC. Differing discussion topics between maternal-fetal medicine and nephrologists suggest a need for PCC standardization and equitable access to multidisciplinary specialists.

## Plain Language Summary

Chronic kidney disease (CKD) is associated with health risks to both the pregnant person and the baby. Therefore, preconception counseling (PCC) is recommended for all patients and is especially important for individuals with chronic disease, like CKD. We surveyed 108 patients who received nephrology care at an urban academic medical center to understand how often and how well PCC is provided. We found that 26% of patients report no receipt of PCC. Maternal-fetal medicine doctors provided more comprehensive PCC and were more likely to discuss medication safety, future childbearing plans, maternal complications, and fetal complications.

## Introduction

Chronic kidney disease (CKD) is defined as kidney damage resulting in an impaired, decreased estimated glomerular filtration rate <60 mL/min/1.73 m^2^ and/or elevated urine albumin-creatinine ratio over 30 for more than 3 months.^1^ Although most patients with stage 1 or 2 CKD experience healthy pregnancies, impaired kidneys may struggle to adjust to the physiological changes of pregnancy. As a result, the risk of adverse maternal and fetal outcomes increases significantly with each subsequent stage of the disease.^2^, ^3^, ^4^, ^5^, ^6^ Examples of adverse maternal events include gestational hypertension, preeclampsia, eclampsia, worsening kidney function, and maternal mortality. Examples of adverse fetal events include premature births, intrauterine growth restriction, low birth weight, and perinatal-neonatal mortality.^2^

Preconception counseling (PCC) is defined as health education and promotion to minimize potential health risks for the pregnant person, fetus, and newborn by optimizing prepregnancy health, managing modifiable risk factors, and educating the pregnant person on practices for a future healthy pregnancy.^7^, ^8^, ^9^ Because a patient’s holistic health status impacts pregnancy, all providers can provide PCC. Furthermore, multidisciplinary PCC provided by primary care providers as well as specialists, including maternal-fetal medicine (MFM) specialists and nephrologists, has been associated with improved outcomes in patients with CKD.^10^

PCC is especially important in pregnant people with known medical comorbid conditions, such as CKD, that may increase their risk of adverse events. Preconception management of CKD includes medication reconciliation and changes, coordinating pregnancy with CKD stability, and coordinating multidisciplinary care by nephrologists and MFM specialists (Table 1).^11^^,^^12^

**Table 1:** Summary of Management Changes Implemented Based on Preconception Counseling in Patients With Kidney Disease

Population	Key Contraindications Identified	Preconception Management Changes
Hypertension/proteinuric Kidney Disease	ACE-I/ARB	Discontinue RAAS inhibitors; transition to pregnancy-compatible antihypertensives; redefine BP targets for maternal-fetal safety
Diabetic nephropathy	ACE-I/ARB; SGLT-2i eg, dapagliflozin; GLP-1 RA	Replace teratogenic agents; initiate pregnancy-safe BP and insulin-based glycemic management; enhance kidney and metabolic monitoring
Glomerular disease	Mycophenolate; teratogenic immunosuppressants	Transition to pregnancy-safe immunosuppression (eg, azathioprine); target disease remission before conception
Polycystic kidney disease	Tolvaptan	Discontinue tolvaptan; optimize BP with pregnancy-safe agents; counsel on hypertensive pregnancy risk
All patients with CKD	Fetotoxic medications; unstable disease	Comprehensive medication reconciliation; time pregnancy during disease stability; coordinate multidisciplinary nephrology, MFM care

ACE-I, angiotensin-converting enzyme inhibitor; ARB, angiotensin II receptor blocker; BP, blood pressure; CKD, chronic kidney disease; GLP-1 RA, glucagon-like peptide-1 receptor agonist; MFM, maternal-fetal medicine; RAAS inhibitors, renin-angiotensin-aldosterone system inhibitors; SGLT-2i, sodium-glucose cotransporter-2 inhibitor.

Although PCC is widely recommended by national organizations, including the Centers for Disease Control and Prevention and the American College of Obstetricians and Gynecologists, there is limited evidence that PCC is provided to people with known CKD. The objective of this study was to identify current utilization and characteristics of PCC, including specific educational topics discussed, among patients with CKD at an urban academic center, to identify targeted opportunities to improve PCC rates and delivery.

## Methods

This cross-sectional study used a patient survey to evaluate self-reported exposure to PCC. The study protocol was approved by the Institutional Review Board of the University of California, Los Angeles (IRB-22-0086).

### Survey design

There were no existing survey instruments relevant to the proposed topic. Thus, this novel survey was developed using established methodological standards, including content validity assessment by experts (C.S.H. and N.N.), evaluation by a trained biostatistician (L.K.), and pilot testing in lay individuals. The survey collected data in 4 main areas: demographics, CKD status, PCC exposure and understanding, characteristics of medical care, and interactions with the health care system (Item S1). Demographic characteristic data included age, race/ethnicity, primary language, relationship status, highest degree of education, housing status, and insurance status. Clinical data for CKD included etiology of CKD, specific CKD therapies, history of dialysis, and history of kidney transplant. Additional clinical data for CKD included reproductive symptoms, such as menstrual irregularities and sexual dysfunction, which may signal hormonal imbalances or fertility barriers associated with CKD progression. Participants also reported exposure to PCC (ie, “Have you had a conversation about reproductive planning with one of the following health care providers before?”), who initiated the conversation; components of PCC received; and self-assessment of concerns and knowledge regarding CKD and pregnancy. Characteristics of medical care, such as interactions with the health care system, including frequency of visits with a nephrologist and an MFM provider, were also recorded. The survey was designed to take about 10 minutes to complete, and patients were compensated with a gift card on completion.

### Survey administration

This survey was distributed via MyChart message between January and May 2024 to all patients aged 18-45 years and assigned female at birth who attended an appointment at a nephrology clinic between November 2023 and February 2024 at an urban academic center. Patients were excluded if they were non-English speakers or had a history of hysterectomy and/or sterilization.

### Statistical analysis

We compared patient demographic characteristics, medical characteristics, and concerns and knowledge of PCC between those who did and did not report receiving PCC using χ^2^ or Fisher exact tests. Among those who received counseling, we also compared PCC components between those counseled with an MFM provider, a nephrologist, or another provider. Other providers included but were not limited to primary care providers, general obstetricians/gynecologists, and infertility endocrinologists. Univariable and multivariable logistic regressions were performed to identify characteristics associated with receipt of PCC.

## Results

**Figure 1:**
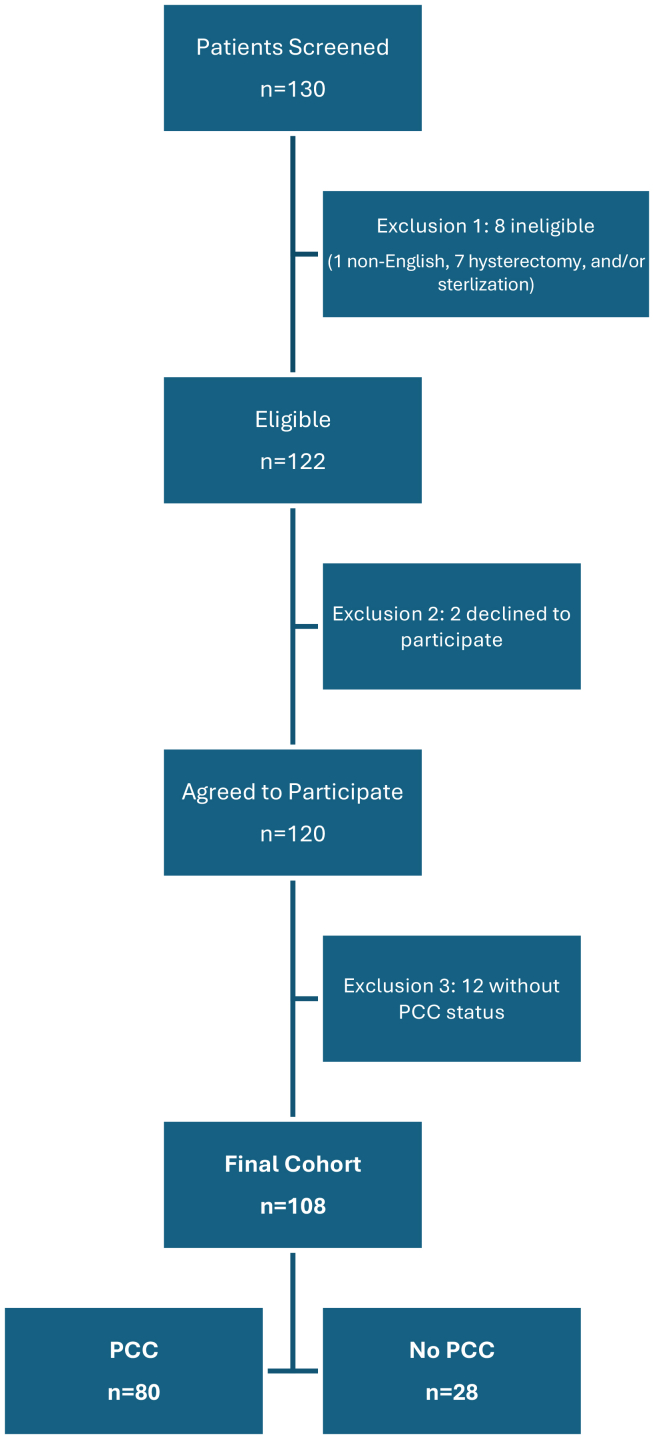
Respondent inclusion and exclusion criteria.

There were 130 surveys distributed to patients assigned female at birth who were of reproductive age and had received care at a University of California, Los Angeles, Division of Nephrology clinic between November 2023 and February 2024. Eight were excluded because they did not meet eligibility criteria (n = 1 non-English speaker and n = 7 with a history of hysterectomy and/or sterilization). Of the 122 surveys distributed to eligible respondents, 120 were completed, achieving a 98% response rate among eligible participants. Of the 120 returned surveys, 12 were excluded because of nonresponse to PCC status, resulting in a final cohort of 108 respondents (Fig 1).

Of this final cohort, 74% (n = 80) of respondents reported receiving PCC. There were no significant differences in demographic characteristics between those who received PCC and those who did not, including age, race/ethnicity, relationship status, education level, housing status, and insurance status (Table 2). Receipt of PCC was also not associated with the severity of kidney disease. The self-reported use of teratogenic CKD medications (ie, methotrexate and mycophenolate mofetil) and active treatment for complications of CKD, such as hypertension, anemia, and electrolyte dysregulation, were not statistically significantly associated with receipt of PCC (Table 3).

**Table 2:** Demographic Characteristics of Respondents Who Did Vs Did Not Report Receiving Preconception Counseling (PCC)

Characteristic		Received Counseling		*P* value
Row %	Total (N = 108)	None (n = 28)	Any (n = 80)	
Age, y, n (%)				0.95^a^
18-34	65 (60.2%)	17 (26.2%)	48 (73.8%)	
35-45	43 (39.8%)	11 (25.6%)	32 (74.4%)	
Race, n (%)				0.55^b^
Asian	27 (25.0%)	6 (22.2%)	21 (77.8%)	
African American	9 (8.3%)	3 (33.3%)	6 (66.7%)	
Native Hawaiian/Pacific Islander	1 (0.9%)	1 (100.0%)	0 (0.0%)	
Other/declined	33 (30.6%)	9 (27.3%)	24 (72.7%)	
White	38 (35.2%)	9 (23.7%)	29 (76.3%)	
Hispanic ethnicity, n (%)				0.31^a^
Yes	49 (45.4%)	15 (30.6%)	34 (69.4%)	
No	59 (54.6%)	13 (22.0%)	46 (78.0%)	
Relationship status, n (%)				0.27^a^
Single	52 (48.1%)	16 (30.8%)	36 (69.2%)	
In a relationship	56 (51.9%)	12 (21.4%)	44 (78.6%)	
Education level, n (%)				0.12^b^
Advanced degree	17 (15.7%)	2 (11.8%)	15 (88.2%)	
Completed college	52 (48.1%)	11 (21.2%)	41 (78.8%)	
Some college	15 (13.9%)	6 (40.0%)	9 (60.0%)	
Completed high school	18 (16.7%)	8 (44.4%)	10 (55.6%)	
Less than high school	6 (5.6%)	1 (16.7%)	5 (83.3%)	
Housing status, n (%)				1.00^b^
Stable housing	107 (99.1%)	28 (26.2%)	79 (73.8%)	
Unhoused	1 (0.9%)	0 (0.0%)	1 (100.0%)	
Insurance, n (%)				0.10^a^
Private or self-pay	58 (54.2%)	11 (19.0%)	47 (81.0%)	
Public	49 (45.8%)	16 (32.7%)	33 (67.3%)	

a χ^2^ test *P* value;.

b Fisher exact test *P* value.

**Table 3:** Characteristics of Underlying Kidney Diagnoses Among Respondents who Did vs Did Not Report Receiving Preconception Counseling

Characteristic		Received Counseling		*P* value
Row %	Total (N = 108)	None (n = 28)	Any (n = 80)	
Duration of CKD, n (%)				0.17^a^
<12 mo	15 (13.9%)	7 (46.7%)	8 (53.3%)	
1-5 y	27 (25.0%)	7 (25.9%)	20 (74.1%)	
6-10 y	28 (25.9%)	4 (14.3%)	24 (85.7%)	
>11 y	36 (33.3%)	9 (25.0%)	27 (75.0%)	
Don't recall	2 (1.9%)	1 (50.0%)	1 (50.0%)	
Underlying cause of CKD, n (%)				
Autoimmune	48 (44.4%)	14 (29.2%)	34 (70.8%)	0.29^b^
Cardiovascular	2 (1.9%)	0 (0.0%)	2 (100.0%)	1.00^a^
Congenital or childhood	7 (6.5%)	2 (28.6%)	5 (71.4%)	0.91^a^
Drug-induced	8 (7.4%)	1 (12.5%)	7 (87.5%)	0.53^a^
Endocrine	15 (13.9%)	3 (20.0%)	12 (80.0%)	0.92^a^
Genetic	23 (21.3%)	5 (21.7%)	18 (78.3%)	0.82^b^
Hypertension	38 (35.2%)	10 (26.3%)	28 (73.7%)	1.00^b^
Infection	9 (8.3%)	1 (11.1%)	8 (88.9%)	0.75^a^
Stone	5 (4.6%)	1 (20.0%)	4 (80.0%)	1.00^a^
Trauma or surgical	1 (0.9%)	0 (0.0%)	1 (100.0%)	1.00^a^
Transplant donor	5 (4.6%)	2 (40.0%)	3 (60.0%)	0.61^a^
Unknown	16 (14.8%)	3 (18.8%)	13 (81.3%)	0.62^a^
Treated with dialysis, n (%)				0.82^a^
Yes - currently treated with dialysis	6 (5.6%)	1 (16.7%)	5 (83.3%)	
No - never treated with dialysis	50 (46.3%)	12 (24.0%)	38 (76.0%)	
No - but prior history of being treated with dialysis	52 (48.1%)	15 (28.8%)	37 (71.2%)	
Received transplant, n (%)	51 (47.2%)	14 (27.5%)	37 (72.5%)	0.73^b^
Frequency of physician visits, n (%)				0.08^a^
Once a year	7 (6.5%)	0 (0.0%)	7 (100.0%)	
Once every 6 mo	17 (15.7%)	2 (11.8%)	15 (88.2%)	
Once every 3 mo	78 (72.2%)	23 (29.5%)	55 (70.5%)	
Less frequently or never	6 (5.6%)	3 (50.0%)	3 (50.0%)	
Current management, n (%)				
BP management: medications	52 (48.1%)	11 (21.2%)	41 (78.8%)	0.54^b^
BP management: lifestyle	28 (25.9%)	5 (17.9%)	23 (82.1%)	0.29^b^
Iron	27 (25.0%)	4 (14.8%)	23 (85.2%)	0.30^b^
Electrolyte management	16 (14.8%)	1 (6.3%)	15 (93.8%)	0.08^b^
Erythropoietin	5 (4.6%)	0 (0.0%)	5 (100.0%)	0.52^a^
Current medications, n (%)				
Mycophenolate mofetil	48 (44.4%)	13 (27.1%)	35 (72.9%)	.80^b^
ACE-inhibitors	13 (12.0%)	1 (7.7%)	12 (92.3%)	.20^b^
ARB	9 (8.3%)	4 (44.4%)	5 (55.6%)	.19^a^
Methotrexate	1 (0.9%)	0 (0.0%)	1 (100.0%)	1.00^a^

ACE, angiotensin-converting enzyme; ARB, angiotensin II receptor blocker; BP, blood pressure; CKD, chronic kidney disease.

a Fisher exact test *P* value.

b χ^2^ test *P* value.

Similarly, most reproductive symptoms were not significantly associated with receipt of PCC, including menstrual irregularities, pain with intercourse, vaginal dryness, or depression. However, loss of libido was positively associated with receipt of PCC, with 84% of respondents who experienced loss of libido reporting receiving PCC (*P* = 0.02) (Table 4).

**Table 4:** Characteristics of Medical Care of Respondents Who Did Vs Did Not Report Receiving Preconception Counseling

Characteristic	Received Counseling			*P* value
Row %	Total (N = 108)	None (n = 28)	Any (n = 80)	
Frequency of physician visits, n (%)				0.079
Once a y	7 (6.5%)	0 (0.0%)	7 (100.0%)	
Once every 6 mo	17 (15.7%)	2 (11.8%)	15 (88.2%)	
Once every 3 mo	78 (72.2%)	23 (29.5%)	55 (70.5%)	
Less frequently or never	6 (5.6%)	3 (50.0%)	3 (50.0%)	
Current CKD management, n (%)				
BP management: medications	52 (48.1%)	11 (21.2%)	41 (78.8%)	0.54
BP management: lifestyle	28 (25.9%)	5 (17.9%)	23 (82.1%)	0.29
Iron	27 (25.0%)	4 (14.8%)	23 (85.2%)	0.30
Electrolyte management	16 (14.8%)	1 (6.3%)	15 (93.8%)	0.08
Erythropoietin	5 (4.6%)	0 (0.0%)	5 (100.0%)	0.52
Current CKD medications, n (%)				
Mycophenolate mofetil	48 (44.4%)	13 (27.1%)	35 (72.9%)	0.80
ACE-inhibitors	13 (12.0%)	1 (7.7%)	12 (92.3%)	0.20
ARB	9 (8.3%)	4 (44.4%)	5 (55.6%)	0.19
Methotrexate	1 (0.9%)	0 (0.0%)	1 (100.0%)	1.00
Reproductive symptoms, n (%)				
Menstrual irregularities	73 (67.6%)	18 (24.7%)	55 (75.3%)	0.66
Depression	56 (51.9%)	13 (23.2%)	43 (76.8%)	0.51
Loss of libido	51 (47.2%)	8 (15.7%)	43 (84.3%)	0.02
Pain with intercourse	30 (27.8%)	6 (20.0%)	24 (80.0%)	0.38
Vaginal dryness	24 (22.2%)	5 (20.8%)	19 (79.2%)	0.52
Reproductive intentions, n (%)				0.14
< 12 mo	3 (2.8%)	0 (0.0%)	3 (100.0%)	
1-5 y	42 (38.9%)	7 (16.7%)	35 (83.3%)	
6-10 y	13 (12.0%)	4 (30.8%)	9 (69.2%)	
11-15 y	7 (6.5%)	4 (57.1%)	3 (42.9%)	
Not interested	43 (39.8%)	13 (30.2%)	30 (69.8%)	

ACE, angiotensin-converting enzyme; ARB, angiotensin II receptor blocker; BP, blood pressure; CKD, chronic kidney disease.

Respondents who planned to conceive within 5 years reported significantly higher rates of PCC than those who planned to conceive in more than 5 years (84% vs 67%, respectively). Discussion of future childbearing varied significantly by provider type and was most frequently reported in conversations with an MFM specialist (72.2%), followed by a nephrologist (48.6%), and then other providers (37.0%) (Table 5).

**Table 5:** Components of Preconception Counseling (PCC) by Provider Type

Counseling Type	Provider Type				
	Total (N = 80)	MFM (N = 18)	Nephrologist (N = 35)	Other provider (N = 27)	*P*-value
Conversation initiation, n (%)					0.64^a^
Patient	47 (59.5%)	13 (72.2%)	21 (60.0%)	13 (50.0%)	
Patient’s family member or partner	3 (3.8%)	0 (0.0%)	2 (5.7%)	1 (3.8%)	
Physician	29 (36.7%)	5 (27.8%)	12 (34.3%)	12 (46.2%)	
Missing	1	0	0	1	
Future childbearing, n (%)					0.04^a^
Yes, have discussed	40 (50.0%)	13 (72.2%)	17 (48.6%)	10 (37.0%)	
No, but would like to discuss	19 (23.8%)	2 (11.1%)	12 (34.3%)	5 (18.5%)	
No, not interested	21 (26.3%)	3 (16.7%)	6 (17.1%)	12 (44.4%)	
Birth control, n (%)					0.17^a^
Yes, have discussed	48 (60.0%)	13 (72.2%)	24 (68.6%)	11 (40.7%)	
No, but would like to discuss	16 (20.0%)	2 (11.1%)	6 (17.1%)	8 (29.6%)	
No, not interested	16 (20.0%)	3 (16.7%)	5 (14.3%)	8 (29.6%)	
Medication safety, n (%)					< 0.001^b^
Yes, have discussed	34 (42.5%)	12 (66.7%)	20 (57.1%)	2 (7.4%)	
No, but would like to discuss	22 (27.5%)	4 (22.2%)	8 (22.9%)	10 (37.0%)	
No, not interested	24 (30.0%)	2 (11.1%)	7 (20.0%)	15 (55.6%)	
Fetal complications, n (%)					0.002^b^
Yes, have discussed	24 (30.0%)	11 (61.1%)	10 (28.6%)	3 (11.1%)	
No, but would like to discuss	29 (36.3%)	5 (27.8%)	15 (42.9%)	9 (33.3%)	
No, not interested	27 (33.8%)	2 (11.1%)	10 (28.6%)	15 (55.6%)	
Maternal complications, n (%)					< 0.001^b^
Yes, have discussed	25 (31.3%)	14 (77.8%)	9 (25.7%)	2 (7.4%)	
No, but would like to discuss	32 (40.0%)	1 (5.6%)	19 (54.3%)	12 (44.4%)	
No, not interested	23 (28.8%)	3 (16.7%)	7 (20.0%)	13 (48.1%)	
Contraception topics discussed					
Barrier, n (%)	42 (71.2%)	13 (76.5%)	17 (65.4%)	12 (75.0%)	0.76^a^
Nonhormonal IUD, n (%)	29 (49.2%)	9 (52.9%)	12 (46.2%)	8 (50.0%)	0.91^b^
Combined Oral Contraceptive, n (%)	32 (54.2%)	9 (52.9%)	16 (61.5%)	7 (43.8%)	0.53^b^
Patch, n (%)	4 (6.8%)	3 (17.6%)	1 (3.8%)	0 (0.0%)	0.19^a^
Ring, n (%)	4 (6.8%)	1 (5.9%)	3 (11.5%)	0 (0.0%)	0.57^a^
Implant, n (%)	12 (20.3%)	2 (11.8%)	6 (23.1%)	4 (25.0%)	0.65^a^
Hormonal IUD, n (%)	23 (39.0%)	7 (41.2%)	13 (50.0%)	3 (18.8%)	0.13^b^
Oral contraceptive: progestin-only, n (%)	12 (20.3%)	4 (23.5%)	7 (26.9%)	1 (6.3%)	0.27^a^
Injection, n (%)	9 (15.3%)	4 (23.5%)	4 (15.4%)	1 (6.3%)	0.43^a^
Sterilization, n (%)	7 (11.9%)	5 (29.4%)	2 (7.7%)	0 (0.0%)	0.032^a^
Other, n (%)	3 (5.1%)	0 (0.0%)	2 (7.7%)	1 (6.3%)	0.61^a^

a Fisher exact test *P* value.

b χ^2^*P* value.

Among respondents who reported receiving PCC, conversations about PCC were more often initiated by the respondent or the respondent’s family member (63%) than by the physician (37%). PCC was most commonly offered by a nephrologist (44%), followed by another provider (34%), and then by an MFM provider (23%). PCC provided by MFM providers was significantly more likely to cover topics of medication safety, maternal complications and fetal complications, and to discuss sterilization as a birth control option (Table 5).

**Figure 2:**
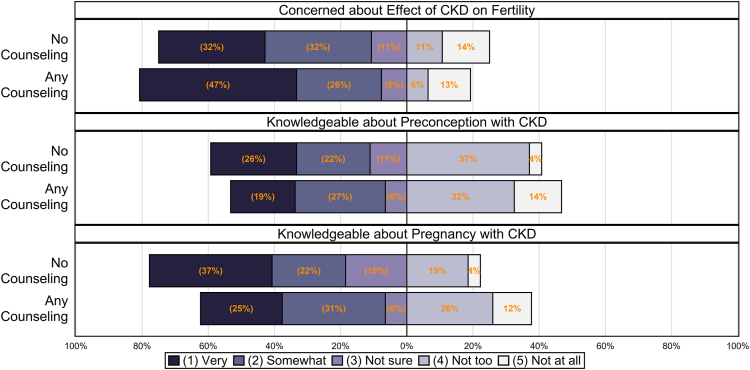
Comparing self-reported concern and knowledge among respondents who did and did not report receiving preconception counseling (PCC).

In terms of the perceived impact of PCC, respondents who reported receiving PCC also reported greater concern about the effect of CKD on fertility but reported feeling less knowledgeable about both preconception and pregnancy with CKD compared with those who reported they did not receive PCC (Fig 2).

## Discussion

To our knowledge, this study is the first to directly compare which specialists provide PCC, what content is emphasized by different providers, and the demographic and medical characteristics of patients with CKD who receive PCC from nephrologists, MFM specialists, and other providers. PCC is an essential and recommended component of care in all patients with comorbid conditions, including CKD. Despite this recommendation, 26% of respondents reported no receipt of PCC. This study was conducted at an urban academic medical center with access to intramural multidisciplinary teams and care. Rates of PCC are even lower nationwide, with average PCC rates of 18%-45% among patients with comorbid conditions and likely exacerbated in underresourced settings.^13^ Reassuringly, no sociodemographic disparities were uncovered in this study, given the payer-agnostic nature of this academic practice. Expansion of PCC should continue to provide equitable care and counseling for all patients with CKD.

One reason for lower rates of PCC is likely the lack of provider-initiated counseling. In this study, most PCC encounters were initiated by patients or their families rather than by physicians. This suggests an opportunity for providers, particularly nephrologists, to proactively provide PCC to patients with CKD. This finding may be due to factors at the system and provider levels. At the system level, PCC is not a routinely covered benefit by many insurance plans.^14^ At the provider level, nephrologists report suboptimal compensation, heavy clinical burden, and rising burnout rates. These factors limit providers from having the time and ability to expand counseling beyond primary complaints to preventative care.^15^

Although health literacy was not measured in this study, respondents who received PCC may have had higher health literacy or a stronger support system to advocate for them to receive PCC.^16^ Furthermore, our finding that respondents who report not receiving PCC reported lower concern but greater knowledge than PCC recipients may suggest false reassurance in the setting of a lack of education. Another possible explanation is that patients who feel knowledgeable or have fewer health concerns do not seek out PCC. These are both missed opportunities because PCC is widely recommended for all individuals with a comorbid condition and is critical for prevention, even in those with less severe disease. Standardizing guidelines for PCC administration for patients and health care models may close gaps in PCC and increase counseling for patients who are not aware of potential complications during pregnancy because of their CKD. On receipt of PCC, additional mental health or support resources may also need to be considered to ensure that patients are equipped to deal with the information.

Our findings identified a need to standardize not only the delivery but also the content of PCC because respondents reported different topics discussed by MFM providers, nephrologists, and other providers. Although PCC was less commonly provided by MFM providers, the PCC content provided by MFM providers was more comprehensive and more likely to include discussions about medication safety, maternal complications, and fetal complications. This suggests a need for standardized education of non-MFM providers to offer similarly comprehensive topics during PCC when access to MFM providers who specialize in providing care through complex pregnancies is limited.

This study had the following limitations. First, because our study included only English-speaking patients at an urban, payor-agnostic academic medical center, our findings may lack generalizability. PCC rates are likely even lower in patients with language discordance. Second, because our study excluded patients with a history of hysterectomy and/or sterilization, this study lacked key information about whether these patients received PCC and whether PCC guided their decision to pursue such a procedure. Third, because this study was based on survey data, we captured self-reported receipt of PCC. Respondents may not have correctly recalled receiving PCC and, therefore, underreported receipt of PCC. Even if this were the case, our results provide practical insight into rates of PCC that were effective and memorable enough for respondents to recall. Finally, the future clinical impact of PCC on pregnancy outcomes and the extent, duration, or depth of PCC were not assessed as part of this study. These questions are worth exploration in future research.

Our findings underscore a critical need to standardize both the delivery and content of PCC in CKD, as respondents reported substantial variability in topics addressed by MFM providers, nephrologists, and other providers. Nephrologists should recognize that comprehensive PCC extends beyond fertility planning to include structured patient education regarding the risk of progression of underlying CKD during and after pregnancy, with counseling tailored to kidney disease etiology, baseline kidney function, and degree of proteinuria. Patients should be informed of the potential for accelerated kidney function decline, hypertensive complications, and disease relapses, as well as the importance of close monitoring during pregnancy and in the postpartum period. PCC should proactively address medication reconciliation and disease-specific management changes, including discontinuation of teratogenic therapies, transition to pregnancy-compatible antihypertensive and immunosuppressive regimens, optimization of glycemic control, and timing of pregnancy during periods of disease stability or remission. Given that care is often fragmented across multiple specialties, standardized PCC should emphasize early multidisciplinary care planning and coordinated postpregnancy follow-up among nephrology, MFM, and primary care to mitigate gaps in surveillance and long-term kidney health.

In conclusion, in nephrology practice, PCC is often initiated by patients or families, highlighting a gap in physician-led preventative care regarding reproductive health. This current practice limits access for those with less awareness or health literacy. Differences in PCC delivery between MFM and other specialist providers underscore the need for standardized cross-disciplinary training. Empowering other providers, including nephrologists, to initiate PCC and promoting multidisciplinary collaboration are critical steps toward ensuring equitable, timely, and comprehensive reproductive care for patients with CKD.

## Supplementary Materials

Supplementary File (PDF) Item S1
