# Supplementary material for: Reproductive Planning and Preconception Counseling in Kidney Disease (REPKID):A Cross-Sectional Survey
Source: Kidney Med. 2026 May 7;8(7):101393. doi: 10.1016/j.xkme.2026.101393 (PMC13279381; doi:10.1016/j.xkme.2026.101393)
Supplement: Supplementary File (PDF) — Item S1 [file mmc1.pdf]

**Item S1:**



# Reproductive Planning in Kidney Disease (REPKID) Study

Thank you for taking time to participate in the "Reproductive Planning in Kidney Disease (REPKID)" Study. This study is an IRB-approved research survey to evaluate the experiences of individuals with current or prior kidney disease in counseling regarding reproductive planning. This survey is anonymous, voluntary and declining to participate involves no penalty.

By participating, you are helping us understand the way in which patients with kidney disease are counseled on future reproductive planning and health. You are agreeing to let us use this data for research and educational purposes. Your responses will remain completely anonymous and data obtained from this study will be stored for future use by the research team. Your responses will not be linked to your medical records, and your providers will not be able to access your responses.

This survey should take no more than 15 minutes.

If you have questions, you may contact:

Niloofar Nobakht MDNephrologynnobakht@mednet.ucla.edu

Christina S. Han, MDMaternal-Fetal Medicinecshan@mednet.ucla.edu

---

You meet eligibility criteria to participate in our survey. Do you agree to participate in this survey?

- ◯ Yes
- ◯ No

## Social Characteristics

| | |
|---|---|
| Are you of Hispanic, Latinx, or Spanish origin? | ◯ Yes<br>◯ No<br>◯ Prefer not to say |
| How would you describe yourself? Select all that apply. | ☐ American Indian or Alaska Native<br>☐ Asian<br>☐ Black or African American<br>☐ Native Hawaiian or other Pacific Islander<br>☐ White<br>☐ Other<br>☐ Prefer not to say |
| What is your current partnership status? | ◯ Single<br>◯ In a relationship<br>◯ Prefer not to say |
| What is the highest degree or level of school you have completed? | ◯ Less than a high school diploma<br>◯ High school degree or equivalent<br>◯ Some college, no degree<br>◯ Associate degree<br>◯ Bachelor's degree<br>◯ Master's degree<br>◯ Professional degree<br>◯ Doctorate |
| What is your housing status? | ◯ Single-family home<br>◯ Multi-family home<br>◯ Apartment<br>◯ Townhouse<br>◯ Condo<br>◯ Co-op<br>◯ Unhoused/transient housing |

**What is your current insurance status? Select all that apply.**

| | Yes | No |
|---|---|---|
| Private insurance | ○ | ○ |
| Public insurance (Medi-Cal/Medicaid, Medicare) | ○ | ○ |
| Not insured | ○ | ○ |
| Private pay | ○ | ○ |

## Kidney Disease

How long ago were you first diagnosed with a kidney disease?

- ◯ < 12 months
- ◯ 1-5 years
- ◯ 6-10 years
- ◯ >11 years
- ◯ Don't recall

**Have you been told by your doctor that one of the following has caused or contributed to your kidney disease? Select all that apply.**

| | Yes | No | I don't know |
|---|---|---|---|
| Autoimmune disease | ◯ | ◯ | ◯ |
| Congenital (At birth) or childhood kidney disease | ◯ | ◯ | ◯ |
| Heart / Cardiovascular disease | ◯ | ◯ | ◯ |
| High blood pressure / Hypertension | ◯ | ◯ | ◯ |
| Genetic disease | ◯ | ◯ | ◯ |
| Infection | ◯ | ◯ | ◯ |
| Endocrine (such as Diabetes) | ◯ | ◯ | ◯ |
| Drug-induced (caused by medication) | ◯ | ◯ | ◯ |
| Kidney stones | ◯ | ◯ | ◯ |
| Trauma-induced kidney damage or surgery / resection | ◯ | ◯ | ◯ |
| Transplant donor | ◯ | ◯ | ◯ |
| Other | ◯ | ◯ | ◯ |

If you selected other, what other cause has contributed to your chronic kidney disease? ________________________________

If you selected autoimmune disease, with which of the following have you been diagnosed?

- ☐ Lupus nephritis
- ☐ IgA nephropathy
- ☐ ANCA-associated vasculitis
- ☐ Henoch-Schonlein purpura (HSP)
- ☐ Anti-GBM vasculitis
- ☐ Polyarteritis nodosa (or large vessel vasculitis)
- ☐ Sarcoidosis
- ☐ Other

If you selected congenital or childhood kidney disease, with which of the following have you been diagnosed?

- ☐ Unilateral renal agenesis
- ☐ Duplicated ureteral system
- ☐ Hydronephrosis (unilateral or bilateral)
- ☐ Vesicoureteral reflux
- ☐ Ureteropelvic junction obstruction
- ☐ Ureterovesical junction obstruction
- ☐ Ureterocele
- ☐ Renal hypoplasia
- ☐ Posterior urethral valves
- ☐ Pelvic or ectopic kidney
- ☐ Cross fused ectopic kidney
- ☐ Other

| | |
|---|---|
| If you selected genetic kidney disease, with which of the following have you been diagnosed? | ☐ Autosomal dominant polycystic kidney disease<br>☐ Autosomal recessive polycystic kidney disease<br>☐ Thin basement membrane disease<br>☐ Gitelman and Bartter syndromes<br>☐ Collagen-related kidney diseases, including Alport syndrome<br>☐ Lowe syndrome<br>☐ Hereditary interstitial kidney disease<br>☐ Tuberous sclerosis<br>☐ Cystinosis<br>☐ Fabry disease<br>☐ Nephronophthisis<br>☐ Other |
| If you selected kidney infection, with which of the following have you been diagnosed in your urinary tract? | ☐ Urinary tract infection<br>☐ Pyelonephritis<br>☐ Sepsis or systemic inflammatory response syndrome (SIRS)<br>☐ Viral infection<br>☐ Bacterial infection<br>☐ Fungal infection<br>☐ Other |
| If you selected endocrine, with which of the following have you been diagnosed? | ☐ Diabetes<br>☐ Hyperparathyroidism<br>☐ Other |
| If you selected drugs, with which of the following have you been diagnosed? | ☐ Aminoglycoside antibiotics or other nephrotoxic antibiotics<br>☐ NSAIDs (over the counter pain or cold medications)<br>☐ Contrast agents<br>☐ Angiotensin converting enzyme inhibitors<br>☐ Other |
| Are you on dialysis? | ◯ Yes - currently on dialysis<br>◯ No - never on dialysis<br>◯ No - but prior history of being on dialysis |
| Have you received a kidney transplant? | ◯ Yes<br>◯ No |
| If you have had a kidney transplant, how many have you received? | ◯ 1<br>◯ 2<br>◯ 3<br>◯ Other |
| On average over the last 5 years, how often do you see your kidney doctor (nephrologist)? | ◯ Once a year<br>◯ Once every 6 months<br>◯ Once every 3 months<br>◯ Less frequently or never |

**What treatment for kidney disease do you receive?**

| | Currently receiving | Not currently receiving, but received in the past | Have never received |
|---|---|---|---|
| Anemia management with iron supplementation | ○ | ○ | ○ |
| Anemia management with erythropoietin | ○ | ○ | ○ |
| Elevated blood pressure management with lifestyle modification | ○ | ○ | ○ |
| Elevated blood pressure management with medication (see examples of medications below) | ○ | ○ | ○ |
| Electrolyte treatment with diet, including low potassium meals, with or without medication | ○ | ○ | ○ |
| Hemodialysis | ○ | ○ | ○ |
| Peritoneal dialysis | ○ | ○ | ○ |

Click here to view examples of blood pressure medications

## Are you taking any of these medications?

| | Currently taking | Not currently taking, but have previously taken | Have never taken |
|---|---|---|---|
| ACE-inhibitor (see examples below) | ○ | ○ | ○ |
| ARB (see examples below) | ○ | ○ | ○ |
| Methotrexate, also known as Rheumatrex or Trexall | ○ | ○ | ○ |
| Mycophenolate mofetil, also known as CellCept, Myfortic, or MMF | ○ | ○ | ○ |

Click here to view examples of ARBs.

Click here to view examples of ACE-inhibitors.

## Are you currently experiencing or have you ever experienced any of the following symptoms?

| | Yes | No |
|---|---|---|
| Depression | ○ | ○ |
| Menstrual irregularities (abnormal periods) | ○ | ○ |
| Diminished vaginal lubrication or lack of cervical mucus | ○ | ○ |
| Pain or discomfort with intercourse / sex | ○ | ○ |
| Loss of libido or sex drive | ○ | ○ |
| Anovulation (not ovulating, not having periods) | ○ | ○ |

## Reproductive Planning

Are you interested in having children in the next:

- ◯ < 12 months
- ◯ 1-5 years
- ◯ 6-10 years
- ◯ 11-15 years
- ◯ Not interested

Has a healthcare provider mentioned birth control options for you in the last 5 years?

- ◯ Yes
- ◯ No

**If a healthcare provider mentioned birth control options in the last 5 years, which of the following have been recommended to you? Select all that apply.**

| | Yes | No |
|---|---|---|
| Barrier, non-hormonal (condoms, diaphragm) | ○ | ○ |
| Non-hormonal IUD (e.g. copper IUD - Paragard) | ○ | ○ |
| Estrogen/progestin ORAL CONTRACEPTIVE | ○ | ○ |
| Estrogen/progestin SKIN PATCH | ○ | ○ |
| Estrogen/progestin VAGINAL RING | ○ | ○ |
| Progesterone only subcutaneous IMPLANT | ○ | ○ |
| Progesterone only IUD (e.g. Mirena, Kyleena, Liletta, Skyla ) | ○ | ○ |
| Progesterone only PILL | ○ | ○ |
| Progesterone only INJECTION | ○ | ○ |
| Sterilization (tubes tied, partner vasectomy) | ○ | ○ |
| Other | ○ | ○ |

**Have you had a conversation about reproductive planning* with one of the following healthcare team members before?**

***Reproductive planning, or preconception counseling, often covers family planning, achieving a healthy body weight, screening and treatment for infectious diseases, updating appropriate immunizations, and reviewing medications for teratogenic effects.**

| | Yes | No |
|---|---|---|
| Primary care physician, including internist and family medicine physician | ◯ | ◯ |
| Kidney doctor (nephrologist) | ◯ | ◯ |
| OB/GYN (general) | ◯ | ◯ |
| OB/GYN (maternal fetal medicine) | ◯ | ◯ |
| OB/GYN (infertility endocrinologist) | ◯ | ◯ |
| Other physician | ◯ | ◯ |
| Other healthcare team member (NP, PA) | ◯ | ◯ |

If you have had a conversation about preconception counseling with a healthcare team member, who initiated the conversation?

◯ You (the patient)
◯ Family member or partner
◯ Physician

You indicated you had a conversation about reproductive planning with a physician not listed above. Please indicate the type of physician with whom you had a conversation about reproductive planning.

__________________________________

**What topics related to reproductive planning have you discussed with your physician? What topics would you like to discuss?**

| | Yes, have discussed | No, but would like to discuss | No, not interested |
|---|---|---|---|
| Future childbearing | ◯ | ◯ | ◯ |
| Appropriate birth control | ◯ | ◯ | ◯ |
| Medication safety during pregnancy | ◯ | ◯ | ◯ |
| Fetal complications (growth restriction, problems with placenta, preterm delivery) | ◯ | ◯ | ◯ |
| Maternal complications | ◯ | ◯ | ◯ |
| Not listed here (please describe in the following question) | ◯ | ◯ | ◯ |

What other topic(s) related to reproductive planning have you previously discussed with your physician?

__________________________________

What other topic(s) related to reproductive planning would you like to discuss with your physician?

__________________________________

How concerned are you about the effect of chronic kidney disease on fertility?

◯ Not at all concerned
◯ Not too concerned
◯ Not sure
◯ Somewhat concerned
◯ Very concerned
◯ Decline to answer

How knowledgeable do you feel about preconception with chronic kidney disease?

◯ Not at all knowledgeable
◯ Not too knowledgeable
◯ Not sure
◯ Somewhat knowledgeable
◯ Very knowledgeable
◯ Decline to answer

How knowledgeable do you feel about pregnancy with chronic kidney disease?

◯ Not at all knowledgeable
◯ Not too knowledgeable
◯ Not sure
◯ Somewhat knowledgeable
◯ Very knowledgeable
◯ Decline to answer

Your e-mail address (for gift card distribution)

____________________________
